# Assessment of Tumor Margin and Heterogeneity of Colorectal Cancer Using Imaging Mass Spectrometry and Image Segmentation

**DOI:** 10.3390/cancers18010169

**Published:** 2026-01-03

**Authors:** Bojan Trogrlić, Ana Bednjanić, Borna Kovačić, Zrinka Požgain, Dario Mandić, Magdalena Kratofil, Jasmina Rajc, Željko Debeljak, Ilijan Tomaš

**Affiliations:** 1 Department of Abdominal Surgery, University Hospital Centre Osijek, J. Huttlera 4, 31000 Osijek, Croatia; bojan.trogrlic@gmail.com (B.T.); abednja@gmail.com (A.B.); kovacicborna@gmail.com (B.K.); zrinkapozgain@outlook.com (Z.P.); 2 Department of Surgery, Faculty of Medicine, Josip Juraj Strossmayer University of Osijek, J. Huttlera 4, 31000 Osijek, Croatia; 3 Clinical Institute of Laboratory Diagnostics, University Hospital Centre Osijek, J. Huttlera 4, 31000 Osijek, Croatia; dario.mandic@gmail.com (D.M.); magdalena.kratofil@gmail.com (M.K.); 4 Department of Laboratory Medicine and Pharmacy, Faculty of Medicine, Josip Juraj Strossmayer University of Osijek, J. Huttlera 4, 31000 Osijek, Croatia; 5 Department of Pathology and Forensic Medicine, University Hospital Centre Osijek, J. Huttlera 4, 31000 Osijek, Croatia; rajcjasmina@gmail.com; 6 Department of Pathological Anatomy and Forensic Medicine, Faculty of Medicine, Josip Juraj Strossmayer University of Osijek, J. Huttlera 4, 31000 Osijek, Croatia; 7 Department of Pharmacology, Faculty of Medicine, Josip Juraj Strossmayer University of Osijek, J. Huttlera 4, 31000 Osijek, Croatia; 8 Department of Oncology, University Hospital Centre Osijek, J. Huttlera 4, 31000 Osijek, Croatia; ilijan.tomas@kbco.hr; 9 Department of Oncology, Faculty of Medicine, Josip Juraj Strossmayer University of Osijek, J. Huttlera 4, 31000 Osijek, Croatia

**Keywords:** colorectal cancer, matrix-assisted laser desorption-ionization mass spectrometry, mass spectrometry imaging, metabolomics

## Abstract

**Background/Objectives**: There is an increasing need for methods that provide improved insight into the molecular basis of colorectal cancer (CRC) and thus a better understanding of its morphological heterogeneity. The objectives of this study were to evaluate the use of imaging mass spectrometry (IMS) to examine tumor margins and gain insight into the molecular heterogeneity of CRC. **Methods**: An observational study involving 10 cases was conducted. Native tissue samples were collected during the subject’s surgery, and consecutively taken tissue sections were immediately prepared for light microscopic and IMS analysis. IMS was performed across the 200–1000 Da mass range, divided into four sub-ranges, using matrix-assisted laser desorption/ionization time-of-flight mass spectrometry (MALDI TOF MS) in both negative and positive modes of ionization. For tumor margin and tissue heterogeneity assessment, image segmentation was used. Segmented MS images were analyzed against the respective light microscopy images of hematoxylin-eosin-stained tissue sections. **Results**: Quantitative analysis of the sample collection indicated that IMS enabled correct recognition of tumor margin in the 800–1000 Da range using binary segmentation. Denary image segmentation depicted tissue heterogeneity in greater detail. The strongest *m*/*z* signals specific to tumor, peritumor, and margins were identified and tentatively annotated: aside from dCTP, all other compounds were patient-specific, indicating interindividual variations in the molecular composition of CRC. **Conclusions**: IMS provides new insights into the morphological and biochemical properties of CRC: binary segmented MS images can clearly depict the tumor margin in the 800–1000 Da range, while denary segmented MS images depict intra- and inter-individual molecular heterogeneity of CRC.

## Simple Summary

There is an increasing need for methods that provide improved insight into the molecular basis of colorectal cancer (CRC) and, in conjunctions with that, a better understanding of its morphological heterogeneity. Hematoxylin and eosin staining does not provide detailed information about the intratumor proteome and metabolome differences that may affect diagnostic evaluation and treatment. Imaging mass spectrometry (IMS) represents a novel technology well-suited for the analysis of tumor tissue that enables phenotyping of malignant diseases. This study aimed to assess whether the greater image information content provided by low mass range IMS can depict CRC heterogeneity and tumor margins.

## 1. Introduction

Colorectal cancer (CRC) is the third most common cancer worldwide and the second and third leading cancer-related cause of death in men and women, respectively [1,2]. In most cases, histological findings are straightforward and leave no doubt about the diagnosis, which can be considered definitive. However, tumor histopathology is limited by its two-dimensional representation of three-dimensional structures, which can lead to the undetected presence of microinfiltrations. Moreover, CRC is morphologically heterogeneous, and current pathologic assessments do not capture this variability on a molecular level. To prevent and reduce tumor understaging and false-negative results, it is necessary to consider what other methods could be used [3]. It is especially important to accurately detect the tumor margins, as they are a crucial factor in determining the type of treatment and the prognosis of the disease [4]. Rapid methods for identifying tumor margins and potential biomarkers have been developed that can expand the spectrum of information compared to conventional histopathological analyses using tissue hematoxylin-eosin (HE) staining [5].

HE staining does not provide detailed information about the intratumor molecular heterogeneity that arises from genetic variations, which are reflected in the proteome and metabolome status. This may affect the treatment response [6]. Imaging mass spectrometry (IMS) represents a novel technology well-suited for the analysis of tumor tissue that enables phenotyping of malignant diseases [7,8]. This includes accurate localization of biomolecules in tissues, providing new insight into the microenvironment of tumors and peritumors [9]. Each pixel is associated with a mass spectrum that reflects the molecular features of the tissue spot, and recorded images contain a vast amount of information. In most cases, the extraction of meaningful and informative images can be achieved through image segmentation methods [10]. TNM status enriched by the information on the exact localization of biomolecules provided by IMS is expected to provide a better understanding of cancer heterogeneity. Recently, some IMS studies on tumor tissue heterogeneity have been published. The CRC microenvironment has been assessed using a combination of IMS and two-photon microscopy [11]. In an effort to reduce the risks of inappropriate treatment response, tumor proteome and metabolome heterogeneity have been analyzed using matrix-associated laser desorption/ionization time-of-flight (MALDI TOF) IMS and ultra-high-performance liquid chromatography coupled to time-of-flight tandem mass spectrometry (UPLC-TOF-MS/MS) [12,13,14].

Most of the previous IMS studies involved proteomic analysis. However, IMS in the low mass range also enabled metabolomic analysis of lung and oral cavity cancers [15,16]. The evolution of spatial metabolomic analysis provides improved insight into the impact of metabolite distribution on cancer biology [17,18]. It is expected that the contents of small molecules significantly vary between the CRC and the peritumor, and even within the tumor mass. If so, despite the CRC heterogeneity, the tumor margin recognition using IMS can be achieved in the lower mass range. This study aimed to assess whether the greater image information content provided by low mass range IMS can depict CRC heterogeneity and tumor margins. MALDI TOF IMS of CRC tumor and peritumor samples from 10 patients was performed in positive and negative ionization modes across the *m*/*z* range of 200 to 1000 Da. Recorded images were segmented using hierarchical clustering, and binary and denary segmented images were evaluated against the corresponding HE stained tissue samples by board-certified pathologists: tumor margin recognition rates, CRC heterogeneity, and tentatively annotated dominant metabolites were analyzed. This study was intended to provide preliminary insight into the potential use of low-mass-range IMS in histopathology. Therefore, advanced pixel classification methods were not evaluated. Instead, a simple yet frequently used method, namely hierarchical clustering, was evaluated. To assess the correlation between conventional histopathological procedures and IMS, H&E staining was used.

## 2. Materials and Methods

### 2.1. Study Design and Materials

An observational study involving a collection of 10 cases was conducted in collaboration with the Department of Abdominal Surgery, the Department of Pathology and Forensic Medicine, and the Clinical Institute of Laboratory Diagnostics at the University Hospital Centre Osijek. The research was approved by the Ethics Committee of the University Hospital Centre Osijek and the Medical Faculty, JJ Strossmayer University of Osijek (R2-7990/2021). The principles of the Declaration of Helsinki were strictly followed: all participants provided informed consent before being included in the study. To be included in the study, patients had to be over 18 years old and had to be treated for CRC, regardless of whether the operation was urgent or elective. The tumor had to be a primary adenocarcinoma. Patients must not have received neoadjuvant chemoradiotherapy, and the tumor had to be larger than 2 cm in size. Incomplete medical documentation and technically defective samples (diameter < 2 cm, wrinkled samples on the slide) were reasons for exclusion.

### 2.2. Methods

#### 2.2.1. Sample Preparation

Native tissue samples from the subjects’ colon were collected during a regular colon resection procedure at the Department of Abdominal Surgery of the University Hospital Centre Osijek. Sampling was carried out immediately after the subjects’ surgery. Native colon tissue samples, which included the subject’s primary tumor, were taken immediately after colon resection surgery. The size of the samples taken was 2–3 cm^3^. They represented the full thickness of the colon wall with surrounding adipose tissue. They contained surrounding both healthy tissue and tissue affected by the tumor process and were stored without additives in plastic tubes at a temperature of −70 °C, to prevent morphological and biochemical degradation until analysis. The remaining part of the resected colon with the primary tumor was used for standard pathohistological analyses. The samples were placed for 20 min in a cryostat at a temperature of −20 °C. Serial sections of the samples were created, and the thickness of a section was 8 µm: two tissue sections were transferred to standard microscopic slides (for histopathological analysis), and two more sections were transferred to indium-tin-oxide (ITO) slides (for IMS).

#### 2.2.2. Histopathology

As part of the standard histopathological processing of the samples, they were fixed in 10% formalin, embedded in paraffin blocks, and then stained with HE. The histopathological findings were based on the ASCO-CAP guidelines for CRC [19]. Optical imaging was conducted using an inverted Zeiss Axiovert 200 M microscope (Carl Zeiss AG, Oberkochen, Germany) and the computer program AxioVision (Carl Zeiss AG, Oberkochen, Germany): halogen light was used, the magnification was set to 5×, and the overlap was set to 20%. In case of unsatisfactory results, the image processing package Fiji ImageJ2 (Version v1.54d) was used [20]. A detailed histopathology report is provided in Supplementary Materials (Table S1).

#### 2.2.3. Sublimation and Recrystallization of the MALDI Matrix

Sublimation and recrystallization of the MALDI matrix on the ITO slide containing tissue sections were performed using the iMLayer (Shimadzu, Kyoto, Japan) device for automated matrix application. For the positive mode, 0.3 g of DHB powder was sublimated for 10 min using the slit tray, and both the normal mode and automatic mode options were used. Recrystallization was carried out with 500 µL of 5% methanol using a thermostat set at 70 °C for 105 s. After recrystallization, the ITO slides were kept in a vacuum desiccator until the samples were completely dry. For the negative mode, 0.3 g of 9-AA powder was sublimated for 10 min using the normal mode and automatic mode options. After that, recrystallization was carried out with 500 µL of 5% methanol for 5 min using a thermostat set to 37 °C. Then, the ITO slides with the preparations were stored in a vacuum desiccator to ensure complete drying.

#### 2.2.4. IMS Procedure

IMS data were collected in positive and negative modes using an UltrafleXtreme MALDI-TOF/TOF (Bruker Daltonics, Bremen, Germany) instrument in the mass range between 200 and 1000 *m*/*z*. The computer programs used for data collection were FlexControl version 3.4 and FlexImaging version 5.0 (Bruker Daltonics, Bremen, Germany). The raster width was 30 µm, and the laser frequency was 2000 Hz. The beam parameter set used was 2_small. The sample rate and digitizer settings were set to 2.50. SNAP was chosen as the preprocessing method. Mass calibration was performed with red phosphorus. The mzML IMS image format produced by the UltrafleXtreme MALDI-TOF/TOF device was converted to the imdx format using ImageReveal MS software (Shimadzu, Kyoto, Japan). The same software was then used to process IMS images. The complete mass ranges of the recorded images were divided into sections: 200–400, 400–600, 600–800, and 800–1000 Da. Considering the heterogeneity of CRC and the importance of tumor margin analysis, we evaluated both ionization modes. Recorded images were preprocessed using total-ion-current (TIC) normalization.

#### 2.2.5. Image Segmentation

Image segmentation relies on pixel coloring based on mass spectra similarity, enabling a clear depiction of the object of interest, i.e., particularly depicting its margins. For image segmentation purposes, a well-described pixel classification method, hierarchical cluster analysis (HCA), was used. It was performed using ImageReveal MS software (Shimadzu Japan). The Euclid distance and single-linkage methods were applied. Only binary (2) and denary (10) clusters were analyzed. To estimate the information content of the image, the size (kb) of the segmented image stored in PNG format was used. At the end of the IMS analysis, the segmented images were visually inspected and interpreted by a pathologist who took part in the IMS image collection and by another independent pathologist.

#### 2.2.6. Tumor Margin and Tissue Heterogeneity Assessment

Binary segmentation was used to depict the tumor margin, while the denary segmentation depicted tumor heterogeneity. Four regions of interest (ROIs) were marked on each binary segmented image by the pathologist: two lay over the tumor, and two lay over the peritumor region. The percentage of color-matching pixels between the tumor and peritumor ROIs was calculated: the margin was correctly localized if less than 10% of peritumor pixels, previously colored by segmentation, matched the color of the tumor. The tumor margin was analyzed in four mass ranges and both ionization modes.

#### 2.2.7. Statistical Analysis and Metabolite Annotation

All statistical calculations were performed using the R statistical programming language [21]. Regression slope differences were determined by a *t*-test, while the matching between IMS- and HE-based margin recognition was assessed by the binomial test. Box and whisker graphs and histograms were generated using the same programming language. The discriminant *m*/*z* signals were tentatively annotated using the Metaspace database [22]. Mass tolerance was set to ±0.1 Da. The pathologist’s assessment was used as the gold standard for determining tumor margins against which the IMS analysis results were compared.

## 3. Results

### Quantitative Analysis of the Complete Sample Collection

**Figure 1:**
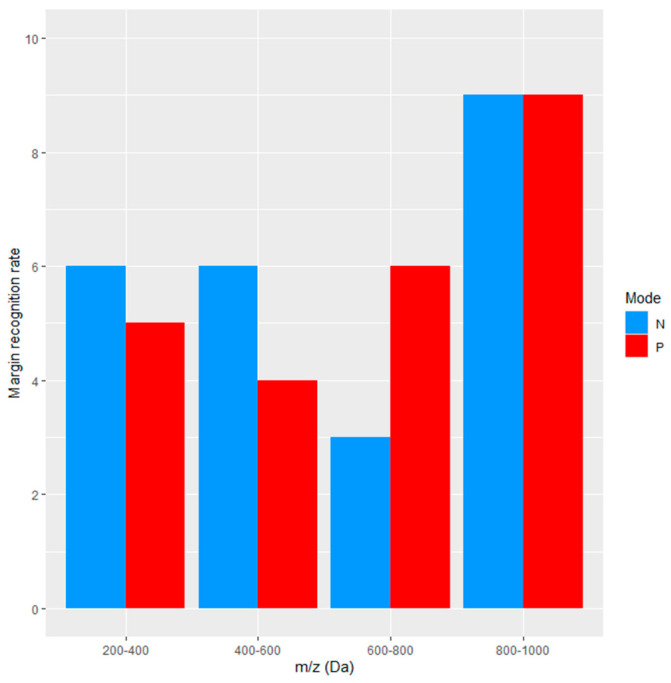
Tumor margin recognition rate for binary–segmented images (*N* = 10).

Across the 800–1000 Da range and binary-segmented images, IMS correctly depicted tumor margins in 9 out of 10 cases (Figure 1), i.e., IMS-based tumor margin recognition was not randomly associated with tumor margins identified by HE staining (*p* = 0.011). For other mass ranges, the tumor margin recognition rate was lower. Due to the high chemical heterogeneity of the analyzed tissues, which was conserved in the denary-segmented images, matching with HE-based tumor margin recognition was not reliable.

**Figure 2:**
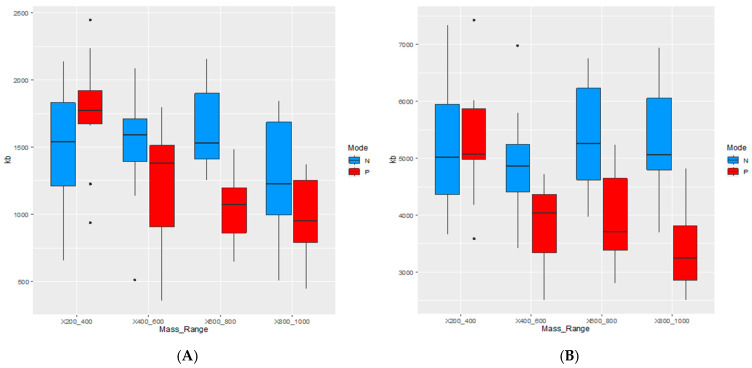
Segmented image information content vs. mass range: (**A**) binary image segmentation; (**B**) denary image segmentation. Boxes were generated using information content of images from all enrolled patients (*N* = 10). Black dots represent outliers.

There is a statistically significant (*p* < 0.005) decreasing trend in the information content of the segmented images recorded in positive mode. In contrast, no such trend is observed in negative mode (Figure 2 and Supplementary Materials, Table S2). In most instances, images recorded in positive mode contain less information in comparison to the images recorded in negative mode. As expected, the information content of images obtained by the denary segmentation is much larger compared to the information content of images produced by the binary segmentation of non–segmented TIC–normalized images. Obviously, denary segmentation depicts tissue heterogeneity in more detail.

**Figure 3:**
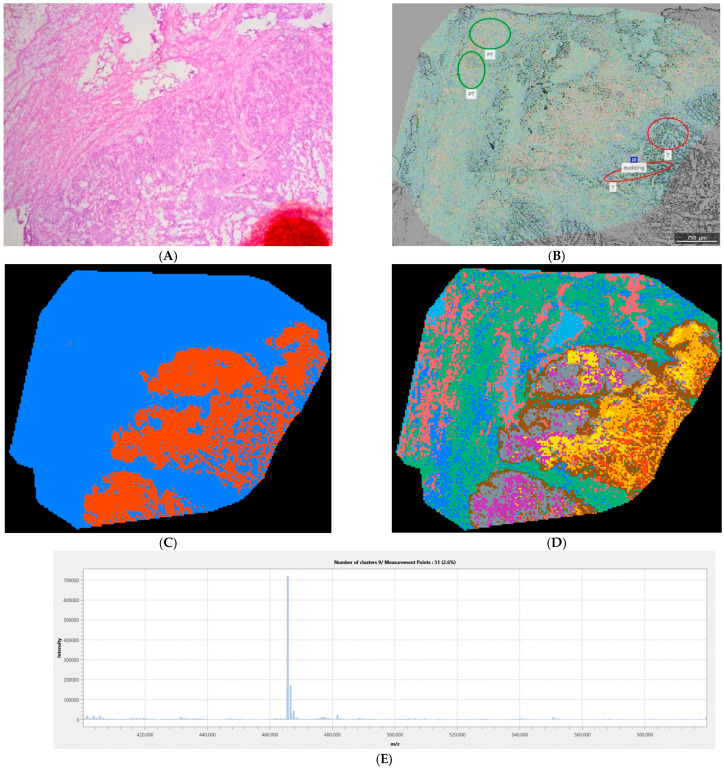
IMS and light microscopy of a representative sample (patient 1): (**A**) HE: image roughly corresponds to the IMS image; (**B**) TIC IMS laid over non-stained light microscopy image: ROIs corresponding to tumor (T), peritumor (PT), and tumor budding were drawn by a certified pathologist; (**C**) Binary segmentation (400–600 Da; negative mode); (**D**) Denary segmentation (400–600 Da; negative mode): tumor margin is colored brown; (**E**) Tumor margin spectrum. The spectra of the other clusters are provided in Supplementary Materials (Figure S1).

Figure 3 provides an example of unequivocal tumor vs. peritumor separation in the lower mass range using negative ionization. Together with the tumor margin, the tumor heterogeneity is clearly presented. In this case, the information content gain provided by denary segmentation even improved the tumor margin depiction. This image is a good example of the results of using binary segmentation in tumor margin recognition. In the case of denary segmentation, a larger number of pixel classes enabled more informative tumor heterogeneity analysis. The dominant *m*/*z* of the tumor margin spectrum is 465.940 Da, which may be tentatively annotated as dCTP.

**Figure 4:**
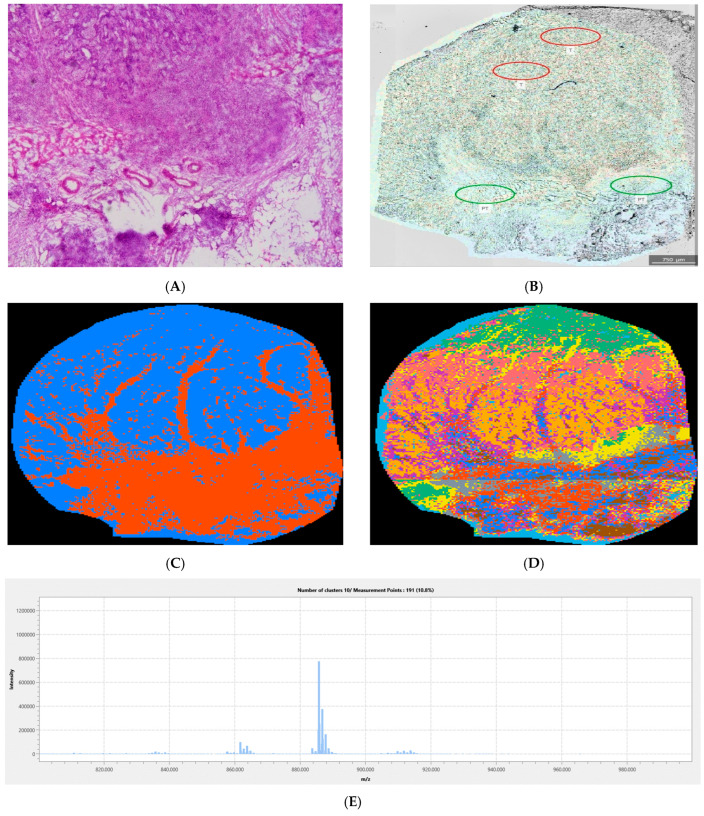
IMS and light microscopy of a representative sample (patient 2): (**A**) HE: image roughly corresponds to the IMS image; (**B**) TIC IMS laid over the non-stained light microscopy image: ROIs corresponding to tumor (T) and peritumor (PT) were drawn by a certified pathologist; (**C**) Binary segmentation (800–1000 Da; negative mode); (**D**) Denary segmentation (800–1000 Da; negative mode): tumor margin is colored grey; (**E**): Tumor margin spectrum. The spectra of the remaining segments are provided in Supplementary Materials Figure S2.

In comparison to Figure 3, Figure 4 more clearly depicts tumor vs. peritumor differentiation in the higher mass range. Both images were produced using the negative ionization mode. In Figure 4, as in Figure 3, the tumor margin is clearly depicted by binary segmentation, but what is particularly interesting is that the tumor margin itself represents a class of its own that is colored gray. In this case, the tumor margin has a biochemical profile distinct from both the tumor and the peritumor tissue, suggesting tumor adaptation to the surrounding tissue. Information content gain, visible in the denary segmentation image, in this case, led to a blurred tumor margin in comparison to the margin depicted in the binary segmented image, which also indicates tumor heterogeneity. The dominant *m*/*z* in the tumor margin spectrum is 885.75 Da, which corresponds to lipid molecules, probably of the triglyceride group.

**Figure 5:**
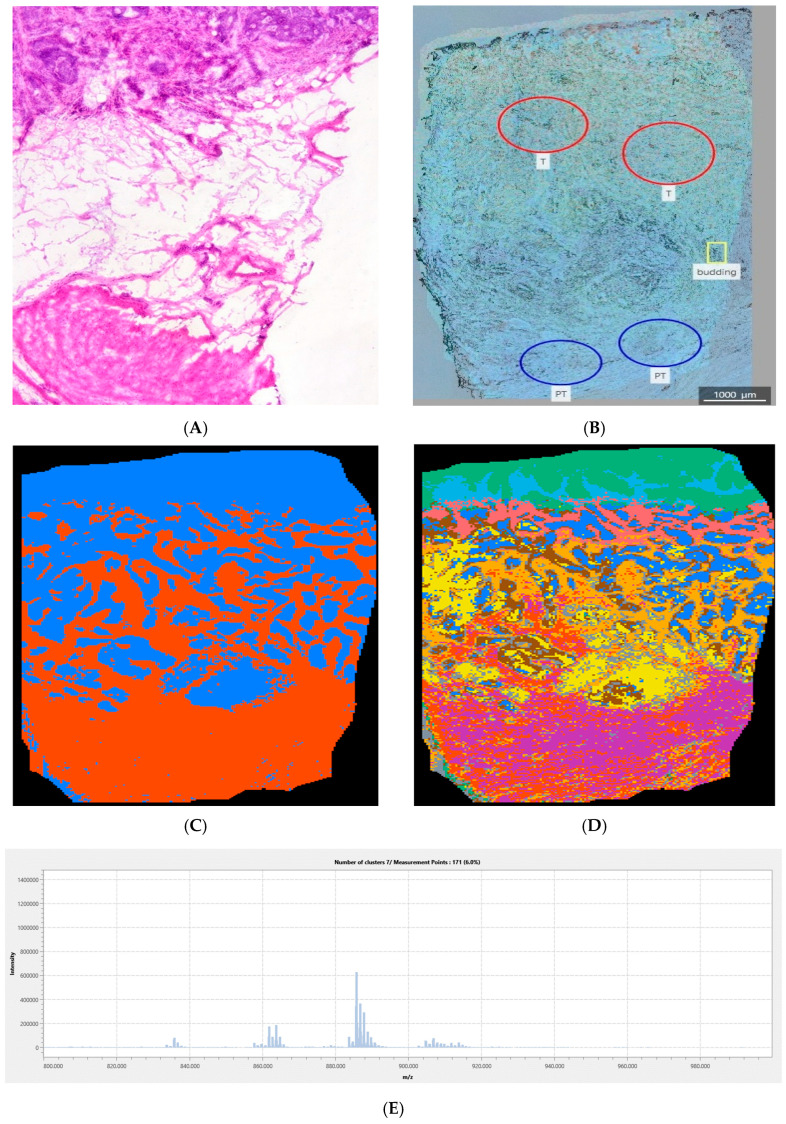
IMS and light microscopy of a representative sample (patient 7): (**A**) HE: image roughly corresponds to the IMS image; (**B**) TIC IMS laid over non-stained light microscopy image: ROIs corresponding to tumor (T), peritumor (PT), and tumor budding were drawn by a certified pathologist; (**C**) Binary segmentation (800–1000 Da; negative mode); (**D**) Denary segmentation (800–1000 Da; negative mode): tumor margin is colored brick-red; (**E**) Tumor margin spectrum. The spectra of the remaining segments are provided in Supplementary Materials (Figure S3).

Figure 5 shows a more complex example: more than two distinct tissue types exist in this sample. However, denary segmentation depicts the tumor margin and the heterogeneity. Even though the binary segmentation depicted the tumor margin, fatty tissue, and the rest of the submucosal tissues were not differentiated in this case, which calls for the use of both binary and denary segmentation. The dominant *m*/*z* in the tumor margin spectrum is 885.75 Da, which corresponds to lipid molecules, probably from the triglyceride group.

## 4. Discussion

### 4.1. Information Content and Margin Detection Rate

Considering the morphological and biochemical heterogeneity of CRC and the increasingly complex histological interpretation of the disease, the research has focused on tumor margin recognition using IMS. Figure 1 does not suggest the existence of any significant differences in terms of tumor margin recognition between negative and positive modes. Images recorded in the 800–1000 Da range, using binary image segmentation, enabled reliable recognition of the tumor margin in 9 out of 10 cases, which was not true for lower masses. Binary segmented images show the best correspondence with HE images and enable the easiest recognition of the tumor margin: the smallest information content is associated with the easiest and most reliable interpretation of the image. In terms of diagnostic utility, the most informative were lipid compounds, which mainly produce signals in the 600–800 and 800–1000 Da mass ranges [23,24]. Possible explanations for the obstacles in depicting the tumor margin at low mass ranges include tumor heterogeneity and the tumor microenvironment (TME) [25,26,27], as well as matrix interference, ion suppression, or metabolite complexity. Given that the TME may be analyzed by IMS, this technique may be used in the search for novel therapeutic targets associated with the TME.

The *m*/*z* range is inversely proportional to the information content of the images recorded in the positive ionization mode, as can be seen in Figure 2. As the correspondence between the pixel coloring and the tissue properties rises, visual differences between tissues in IMS images become sharper, and thus the tumor margin becomes more clearly visible. More uniform mass spectra generate uniform pixel groups that are associated with a decrease in information content. Information content describes the features that can be extracted from an image and is influenced by characteristics such as texture, intensity variation, and spatial relationships between pixels [28]. The information content at lower masses is high, probably due to noise, which is indicated in Figure 2. Despite the high information content in the negative mode, the margin recognition rate is maintained, even in the case of denary segmentation (Figure 2). Visual inspection (Figure 3, Figure 4 and Figure 5) suggests that the negative mode, though associated with higher information content, provides more reliable tumor margin recognition: the information contained in the images recorded in the negative mode is not associated with noise, but comes from better correspondence between tissues and their chemical composition, as reflected in the associated mass spectra.

### 4.2. Impact of Tissue Heterogeneity

To qualitatively assess the performance of the described IMS approach to recognition of the tumor margin and its heterogeneity, several representative images were selected (Figure 3, Figure 4 and Figure 5). Since the information content decreases with increasing mass and the tumor margin was more reliably depicted in higher mass ranges (Figure 1), the mass ranges of 600–800 and 800–1000 Da were presented in the given figures. Due to tissue complexity, both binary and denary IMS image segmentations were analyzed. In this study, only the mean mass spectra of image segments comprising the tumor margin were analyzed; analysis of the complete tumor tissue raw mass spectra was not performed, as the aim of this study was not to determine biomarkers but to assess tumor margins and heterogeneity. By examining the mass spectra of the tumor and peritumor regions of all patients, very few common *m*/*z* values were found. This indicates interindividual differences in the chemical properties of the tumor, the tumor margin, and the peritumor region, which highlights the importance of a personalized approach to each patient. Despite this, for most patients, it was possible to clearly depict the margin by applying segmentation to IMS images.

Figure 3D provides the tumor tissue region that is divided into multiple classes: each tumor tissue class is depicted by a different color corresponding to a specific average mass spectrum. Multiple tumor tissue classes indicate tumor heterogeneity, i.e., different biochemical profiles existing in different parts of the tumor. The ad hoc selected number of 10 segments may be too large and may lead to the analysis of noise instead of analysis of true biochemical differences in the parts of the tissue analyzed. However, if the selection of 10 segments is inappropriate, blurry tissue structures with fuzzy borderlines would be expected: visual inspection of fine features like tumor margins, clearly depicted in denary images (Figure 3, Figure 4 and Figure 5), shows that the number of 10 segments is not excessive, i.e., it closely reflects the differences in biochemical profiles. On the other hand, the tumor margin is more easily recognized in a binary segmented image (Figure 3C). This example shows that, for better tumor margin recognition by visual inspection, it is better to use a smaller number of pixel classes, but for tumor heterogeneity analysis, it is better to increase the number of pixel classes, especially if more tissue types are expected to exist in the sample. Pixels colored brown clearly define the tumor margin and distinguish it from the rest of the tumor and peritumor tissue. The dominant *m*/*z* value in the corresponding mass spectrum is 465.94 Da, which may be tentatively annotated as deoxycytidine triphosphate (dCTP). *m*/*z* 465.94 Da appeared as a tumor feature in samples from almost all patients. The human dCTP pyrophosphatase 1 (DCTPP1) is a newly identified pyrophosphatase regulating the cellular nucleotide pool. Feng et al. pointed to the importance of inhibitors of the regulation of metabolic reprogramming by DCTPP1 and concluded that such inhibitors could have an effect in the treatment of CRC [29]. The results presented in Figure 3 are consistent with the proposed dCTP metabolism targeting.

As shown in Figure 4, IMS images obtained in the higher mass range (800–1000 *m*/*z*) reveal tumor tissue penetration into healthy tissue: the dominant *m*/*z* signal for distinguishing tumor from peritumor tissue was 885.75 Da (Figure 4 and Figure 5). In addition to depicting tumor penetration and tumor edges, it is also noticeable that, as shown in Figure 3, more colors, i.e., different mass spectra, belong to tumor tissue than to peritumor tissue, indicating tumor heterogeneity. The *m*/*z* signals of tumor and peritumor tissue (Supplementary Materials, Figure S2), especially those coming from the spectra of orange pixels, show that there is a difference between the signal intensity of the tumor and the peritumor area. In the selected *m*/*z* range, the tumor and peritumor areas contain biomolecules of the same or similar origin. However, the content of these biomolecules differs between the tumor and the peritumor, which enables the depiction of the tumor edge, i.e., tumor penetration into healthy tissue. It is notable that the intensity at 864.15 Da *m*/*z* (Supplementary Materials, Figure S2) coincides with the transition from peritumoral to tumor tissue and is significantly higher in tumor tissue. The area colored red (Figure 4D) in the denary segmented image is particularly interesting: it is a peritumoral region. Still, it has a high intensity of the previously mentioned signal, which suggests a possible alteration of the peritumoral tissue. This kind of alteration in the chemical composition of tissues cannot be detected using HE staining. The gradient of signal intensity at 864.15 Da corresponds to malignant transformation in the two cases presented (Figure 4 and Figure 5). Oppenheimer et al. have shown that molecular and histological tumor margins do not necessarily coincide in renal cell carcinoma cases [30]. The same may be expected to occur in CRC, especially in aggressive forms: the IMS method presented here may be an approach to faster, more informative visualization of tumor margins that accounts for tumor heterogeneity.

As an example of a more complex case (Figure 5), the coexistence of three or more tissue types is shown. Figure 5C indicates that IMS assigned the same color to fat and the rest of the peritumoral tissue; this suggests that binary classification may not be appropriate for reliable tumor margin recognition in all cases. To more realistically visualize the tumor margin, it is necessary to analyze both binary and denary classifications. Mass spectra obtained by binary segmentation of the image corresponding to different parts of the peritumor or tumor are similar to each other. For this reason, it is important to do a binary and denary classification: the margin can be displayed and interpreted more reliably, which is not guaranteed if only a binary segmented image is analyzed. Analyzing Figure 5A, it gives the impression of a simple distribution of different tissues that are separated, but when compared to Figure 5D, it is evident that this image, which contains the denary classification, provides considerably more information in the biochemical profiles of these tissues and shows that there is actually no clear boundary between these tissues. This depicts a great impact of the tumor tissue on the surrounding peritumoral tissue. The most important differences in mass spectra in the denary images are seen in the transition area along the peritumor to the tumor, i.e., in the tumor margin.

As stated in the Introduction, IMS has been shown to have potential clinical value, as it enables the evaluation of tumor heterogeneity and provides additional value over conventional pathohistological analyses. It would be particularly interesting to perform IMS analyses of tumor budding, which is known to affect the disease’s prognosis [31,32,33,34]. Patients who suffer from rectal cancer and have a histopathological complete response after chemoradiotherapy are a potential group that could benefit significantly from IMS. A patient with a complete response after chemoradiotherapy for rectal cancer is a candidate for the “watch and wait” approach, which enables the patient to be treated without a permanent colostomy or radical surgery. However, this type of treatment currently depends on whether it is possible to recognize the complete histopathological response adequately and on whether it is possible to predict it [35]. Here, IMS offers a potential solution.

At the end, the major limitations of this study should be mentioned: the study included a small number of subjects, while MS2 or other more reliable annotation methods were not applied. Since only MS1 analysis was performed, it cannot be determined with certainty which molecular species correspond to the selected *m*/*z* values. Also, this is a preliminary investigation, and future research should expand the sample size to validate the findings. Other IMS techniques, like desorption electrospray ionization imaging mass spectrometry (DESI-IMS) or secondary-ion mass spectrometry (SIMS), may be used. In this study, MALDI settings were not adapted for the tumor budding detection, which would improve the diagnostic utility of the analysis [34]. Studies that will follow may also focus on applying advanced classification algorithms and comparisons of IMS vs. immunohistochemical procedures. An ad hoc approach to the selection of the number of image segments may be replaced by advanced algorithms that offer a statistically sound selection of the number of image segments. It is expected that this will provide a more reliable insight into tumor heterogeneity. Also, the performance of IMS for tumor margin recognition may be more rigorously evaluated against immunohistochemical procedures. Despite its limitations, this study offers preliminary insight into IMS-based tumor heterogeneity and associated tumor margin recognition.

## 5. Conclusions

Using binary image segmentation, IMS can clearly depict the tumor margin, at least in the 800–1000 Da mass range: tumor heterogeneity can blur the margin, especially in the denary image segmentation. Still, denary segmentation is required for the analysis of samples containing three or more tissue types. Only a few *m*/*z* signals were shared by two or more CRC patients, indicating high interindividual variability, at least in the low mass ranges. In light of CRC heterogeneity, image segmentation and IMS offer new insights into the morphological and biochemical properties of tissues and thus enable an individualized approach to patients with CRC.

## Data Availability

The data that support the findings of this study are available from the corresponding author upon reasonable request.

## Supplementary Materials

The following supporting information can be downloaded at https://www.mdpi.com/article/10.3390/cancers18010169/s1: Table S1. Detailed histopathology report; Table S2. Statistical analysis of the segmented image’s information content; Figure S1. Patient 1 sample spectra; Figure S2. Patient 2 sample spectra; Figure S3. Patient 3 sample spectra.

## Abbreviations

The following abbreviations are used in this manuscript:

CRC	colorectal cancer
HE	hematoxylin-eosin
IMS	imaging mass spectrometry
MALDI TOF	matrix-associated laser desorption/ionization time-of-flight
UPLC-TOF-MS/MS	ultra-high-performance liquid chromatography coupled to time-of-flight tandem mass spectrometry
ITO	indium-tin-oxide
ROI	region of interest
TME	tumor microenvironment
dCTP	deoxycytidine triphosphate
dCTPP1	dCTP pyrophosphatase 1
DESI-IMS	desorption electrospray ionization imaging mass spectrometry
SIMS	secondary-ion mass spectrometry
