# Supplementary material for: Assessment of Tumor Margin and Heterogeneity of Colorectal Cancer Using Imaging Mass Spectrometry and Image Segmentation
Source: Cancers (Basel). 2026 Jan 3;18(1):169. doi: 10.3390/cancers18010169 (PMC12785023; doi:10.3390/cancers18010169)
Supplement: Supplementary file 1 [file cancers-18-00169-s001.zip › cancers-4042604-supplementary.pdf]

SUPPLEMENTARY MATERIALS

Assessment of Tumor Margin and Heterogeneity of Colorectal Cancer Using Imaging Mass Spectrometry and Image Segmentation

Contents:

1. Table S1. Detailed histopathology report
2. Table S2. Statistical analysis of the segmented image’s information content
3. Figure S1. Patient 1 sample specta
4. Figure S2. Patient 2 sample spectra
5. Figure S3. Patient 3 sample spectra

1. Table S1. Detailed histopathology report

Patient	Histological grading	pTNM	Tumor budding	Perineural invasion	Lymphovascular Invasion (LVI)	Tumor Infiltrating Lymphocytes (TIL)	KRAS	NRAS	BRAF	Microsatellite Instability (MSI)
1	G3	T4aN1bM0	BD2	pos	pos	low	WT	WT	Mutant (V600E/D)	deficient
2	G2	T3N0M0	BD2	neg	neg	medium	WT	WT	WT	deficient
3	G2	T3N0M0	BD2	neg	neg	medium	WT	WT	WT	proficient
4	G2	T3N2aM0	BD2	neg	neg	low	WT	WT	WT	proficient
5	G2	T3N0M0	BD2	neg	neg	medium	WT	WT	WT	proficient
6	G2	T3N1aM0	BD3	pos	pos	high	Mutant (G12A)	WT	WT	proficient
7	G2	T3N0M0	BD1	neg	neg	low	WT	WT	WT	proficient
8	G2	T3N2aM0	BD1	neg	neg	medium	Mutant (G12V)	WT	WT	proficient
9	G2	T2N0M0	BD1	neg	neg	medium	Mutant (G12A)	WT	WT	proficient
10	G3	T3N0M0	BD1	neg	neg	high	WT	Mutant (G12D)	WT	deficient

2. Table S2. Statistical analysis of the segmented image information content

Statistical significances: FDR corrected Mann-Whitney U test

Segmentation	P_200 vs N_200	P_400 vs N_400	P_600 vs N_600	P_800 vs N_800
Binary	0.316	0.317	0.016	0.317
Denary	0.770	0.129	0.041	0.039

P and N stand for ionization modes; _200, _400_600 and _800 mark m/z ranges: 200 – 400 Da, 400 – 600 Da, 600 – 800 Da and 800 – 1000 Da, respectively.

Statistical significances: T-test of the regression slope (ax+b, a = 0)

Segmentation	P	N
Binary	< 0.001	0.3323
Denary	< 0.001	0.129

P and N stand for ionization modes

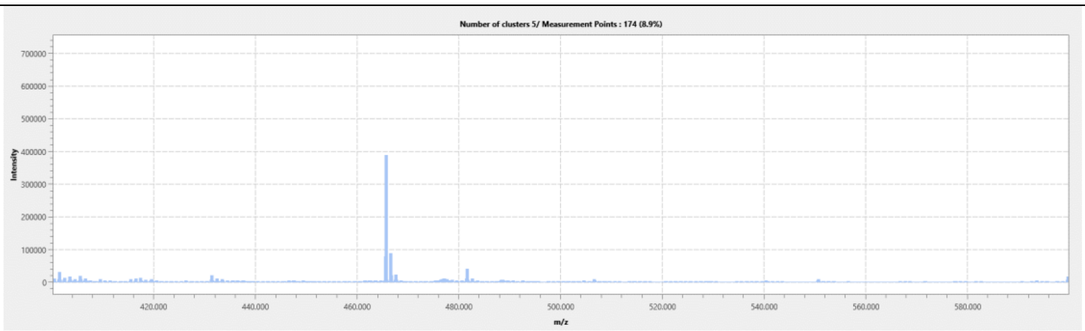
Number of clusters 5/ Measurement Points : 174 (8.9%)
Intensity
700000 600000 500000 400000 300000 200000 100000 0
420.000 440.000 460.000 480.000 500.000 520.000 540.000 560.000 580.000
m/z

1

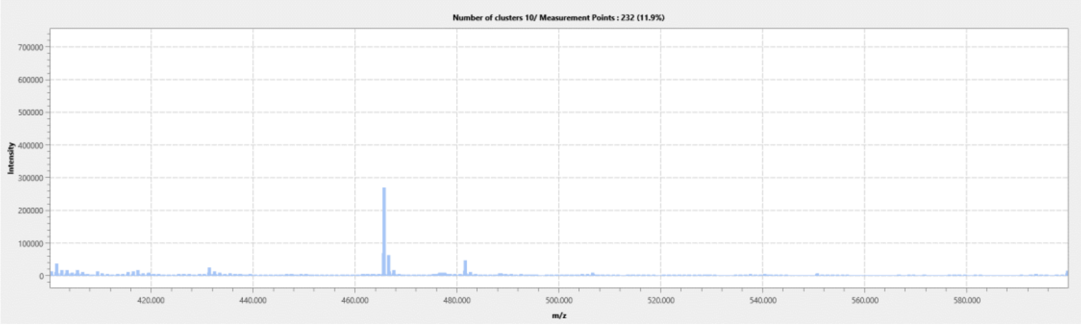
Number of clusters 10/ Measurement Points : 232 (11.9%)
Intensity
700000 600000 500000 400000 300000 200000 100000 0
420.000 440.000 460.000 480.000 500.000 520.000 540.000 560.000 580.000
m/z

2

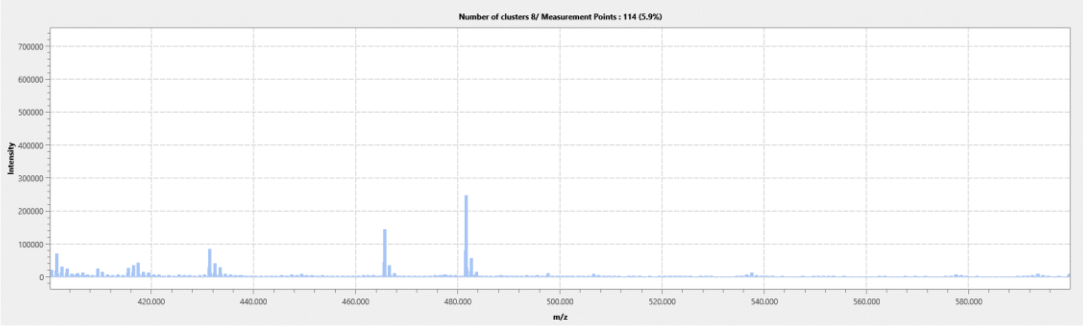
Number of clusters 8/ Measurement Points : 114 (5.9%)
Intensity
700000 600000 500000 400000 300000 200000 100000 0
420.000 440.000 460.000 480.000 500.000 520.000 540.000 560.000 580.000
m/z

3

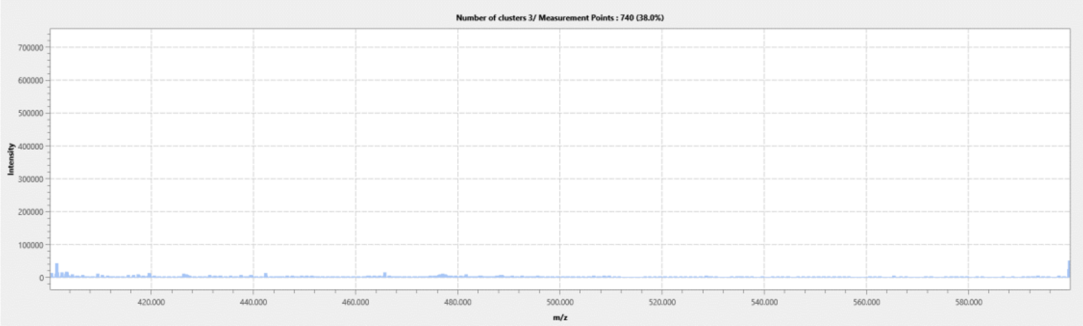
Number of clusters 3/ Measurement Points : 740 (38.0%)
Intensity
700000 600000 500000 400000 300000 200000 100000 0
420.000 440.000 460.000 480.000 500.000 520.000 540.000 560.000 580.000
m/z

4

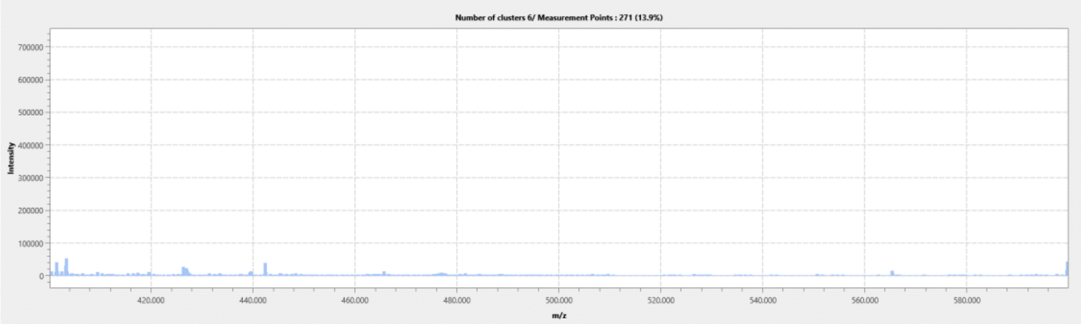
Number of clusters 6/ Measurement Points : 271 (13.9%)
Intensity
700000 600000 500000 400000 300000 200000 100000 0
420.000 440.000 460.000 480.000 500.000 520.000 540.000 560.000 580.000
m/z

5

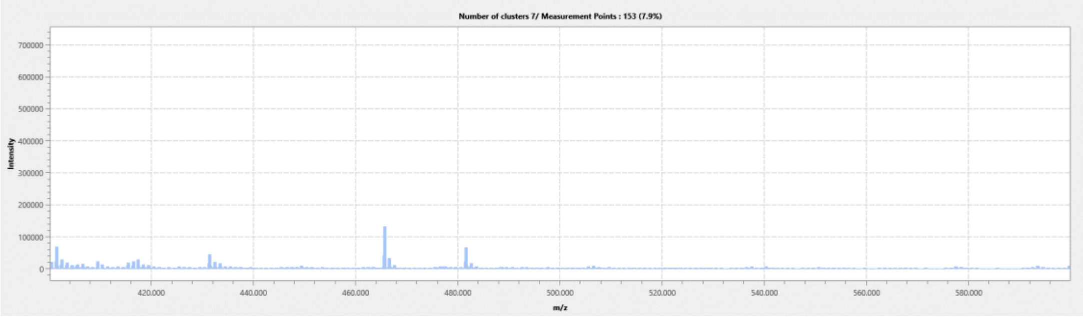
Number of clusters 7/ Measurement Points : 153 (7.9%)
Intensity
700000 600000 500000 400000 300000 200000 100000 0
420.000 440.000 460.000 480.000 500.000 520.000 540.000 560.000 580.000
m/z

6

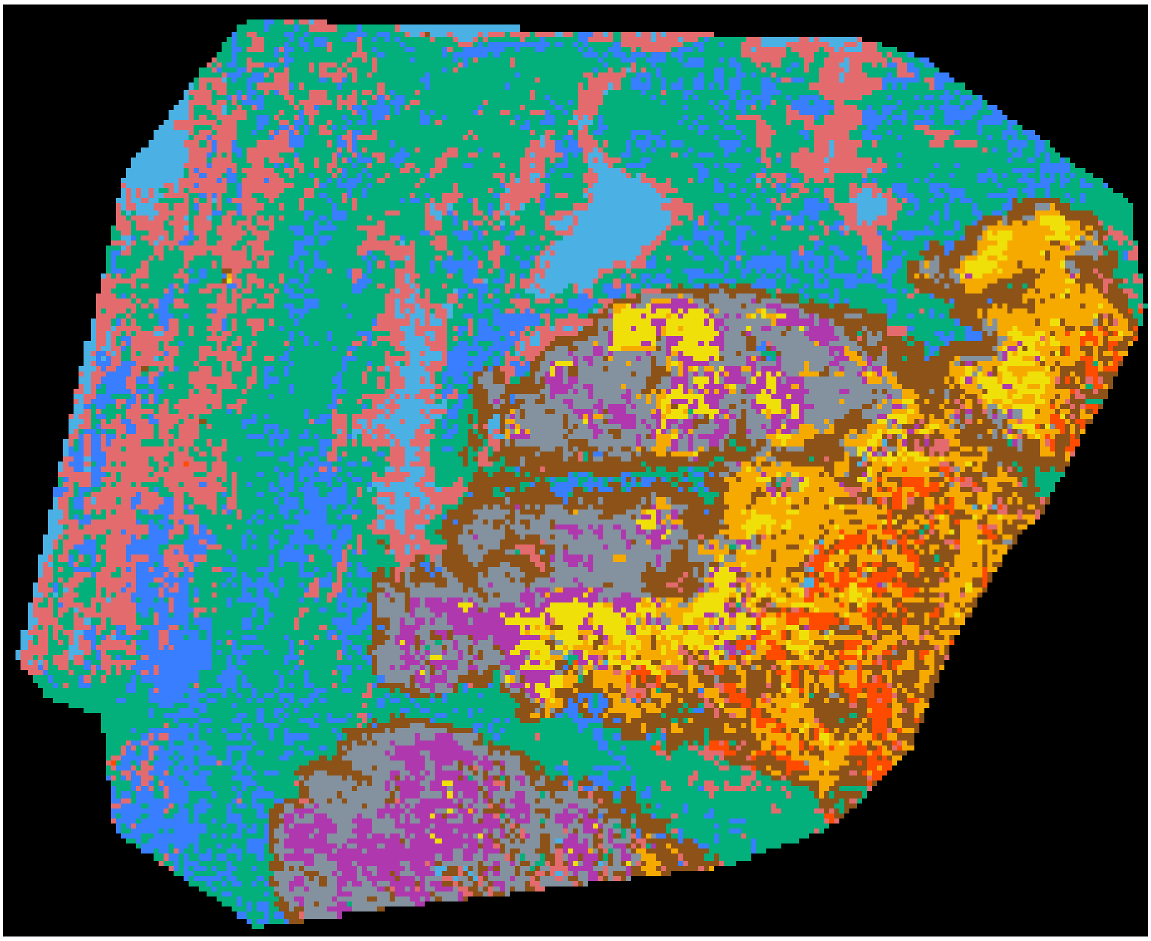

Figure S1. Sample spectra (Patient 1) and denary segmentation picture (400-600 Da; negative mode)

Tumor pixel coloring (1-3): Orange, Grey, Yellow

Peritumor pixel coloring (4-6): Green, Brick, Blue

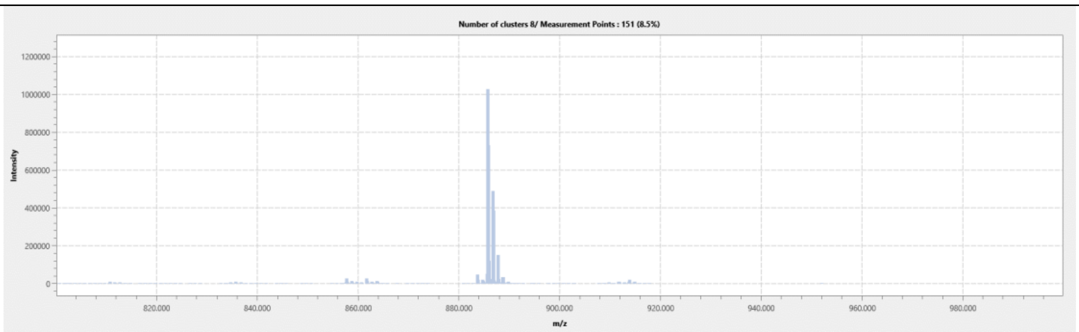
Number of clusters 8/ Measurement Points : 151 (8.5%)
Intensity
m/z
1

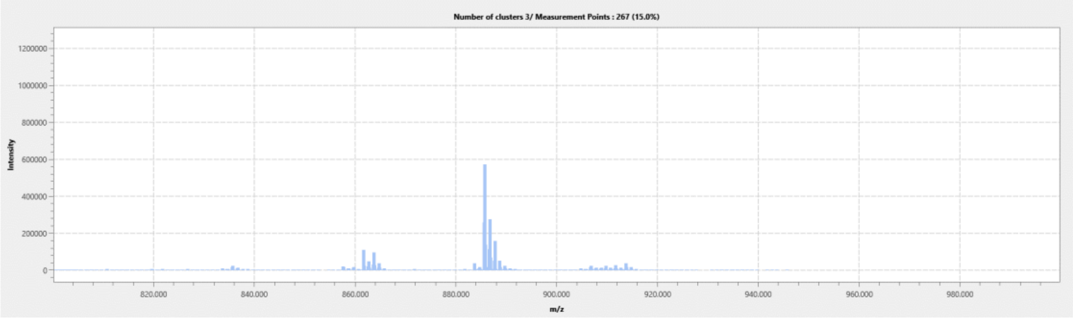
Number of clusters 3/ Measurement Points : 267 (15.0%)
Intensity
m/z
2

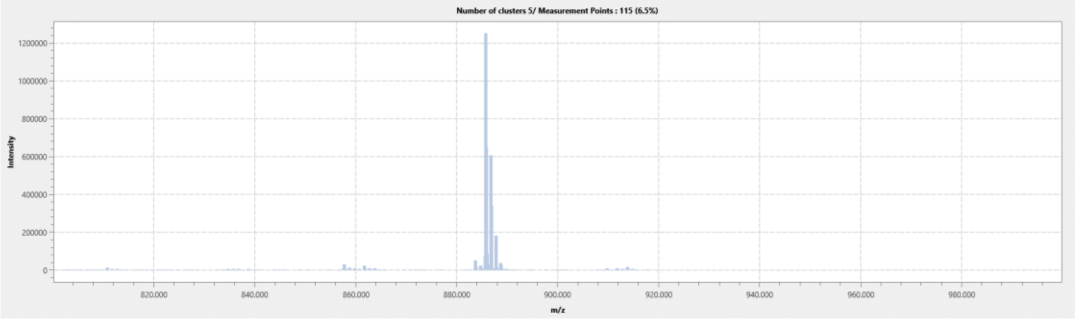
Number of clusters 5/ Measurement Points : 115 (6.5%)
Intensity
m/z
3

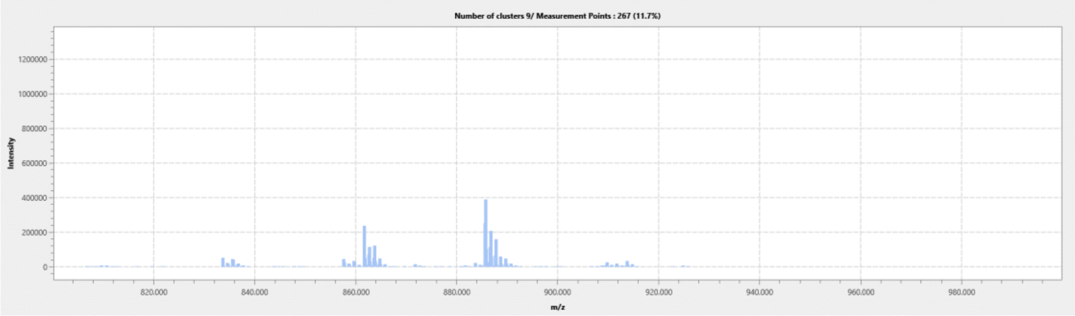
Number of clusters 9/ Measurement Points : 267 (11.7%)
Intensity
m/z
4

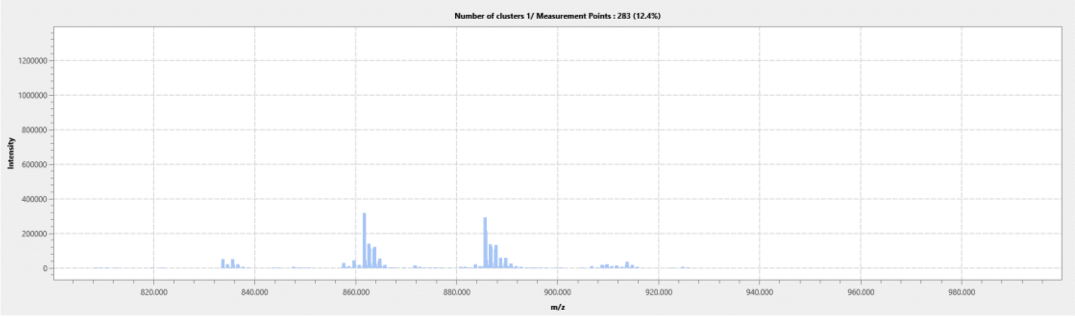
Number of clusters 1/ Measurement Points : 283 (12.4%)
Intensity
m/z
5

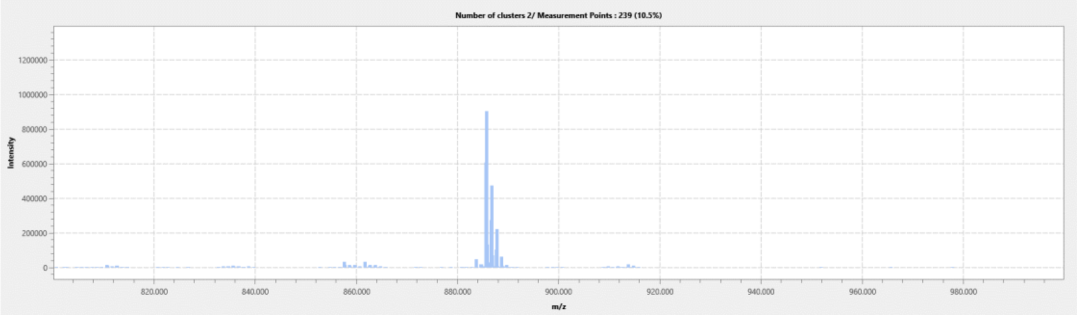
Number of clusters 2/ Measurement Points : 239 (10.5%)
Intensity
m/z
6

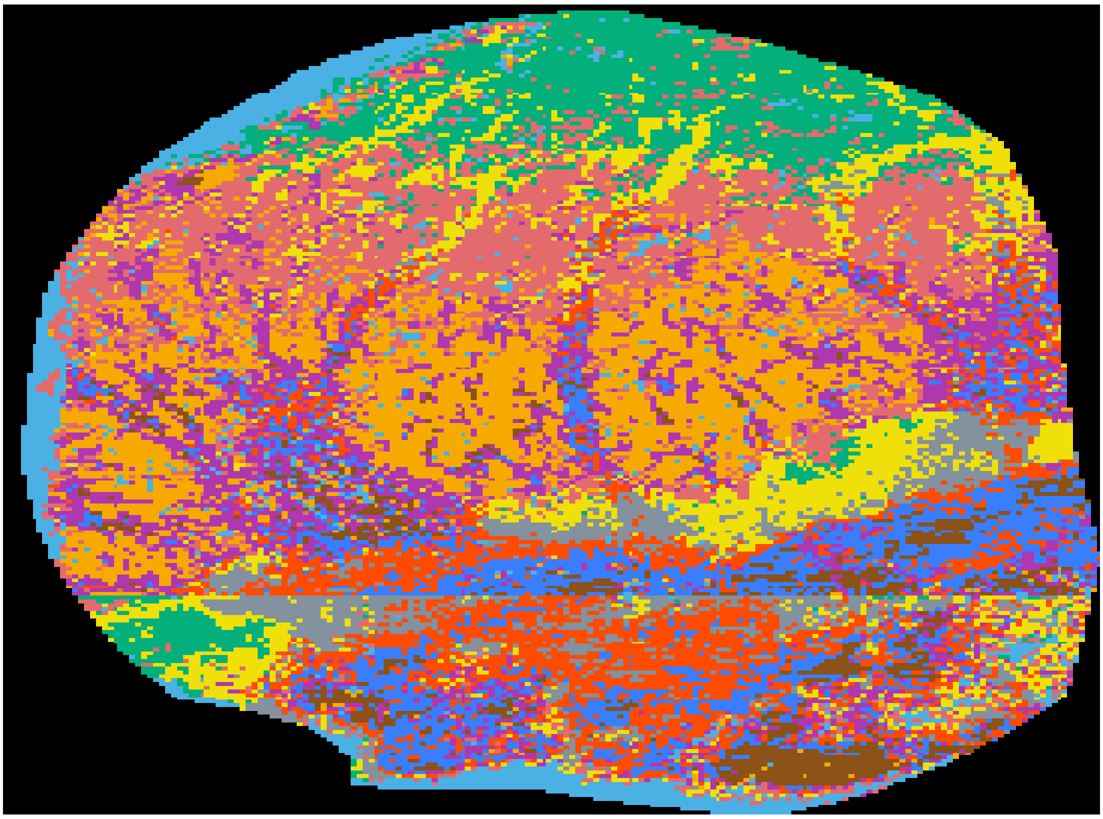

Figure S2. Sample spectra (Patient 2) and denary segmentation picture (800-1000 Da; negative mode)

Tumor pixel coloring (1-3): Brick, Green, Orange

Peritumor pixel coloring (4-6): Red, Blue, Brown

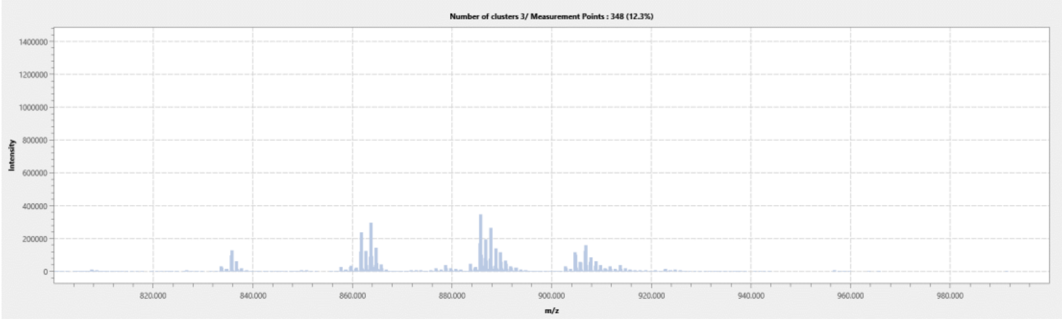
Number of clusters 3/ Measurement Points : 348 (12.3%)
Intensity
m/z
1

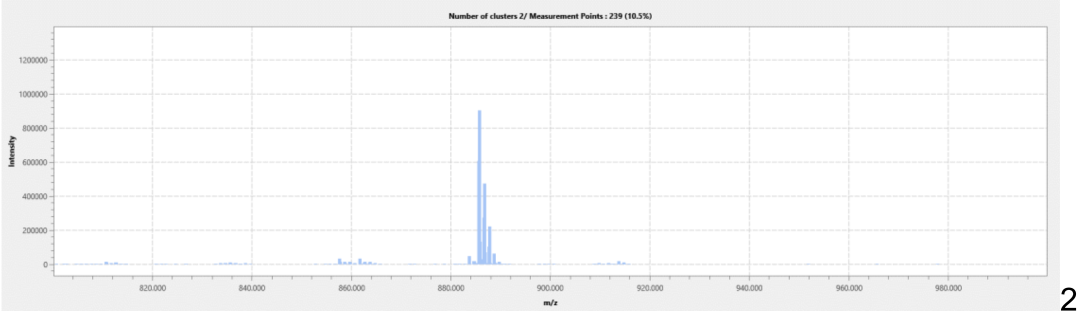
Number of clusters 2/ Measurement Points : 239 (10.5%)
Intensity
m/z
2

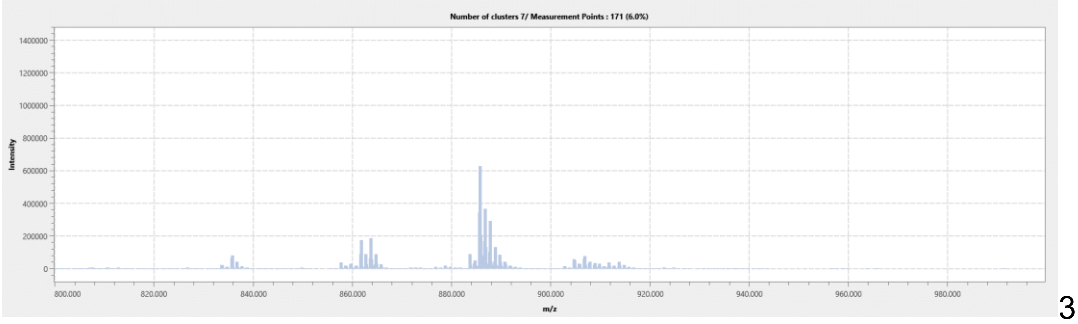
Number of clusters 7/ Measurement Points : 171 (6.0%)
Intensity
m/z
3

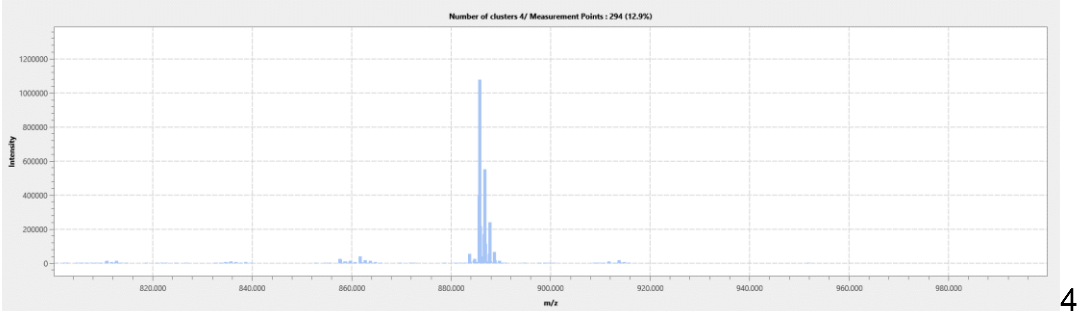
Number of clusters 4/ Measurement Points : 294 (12.9%)
Intensity
m/z
4

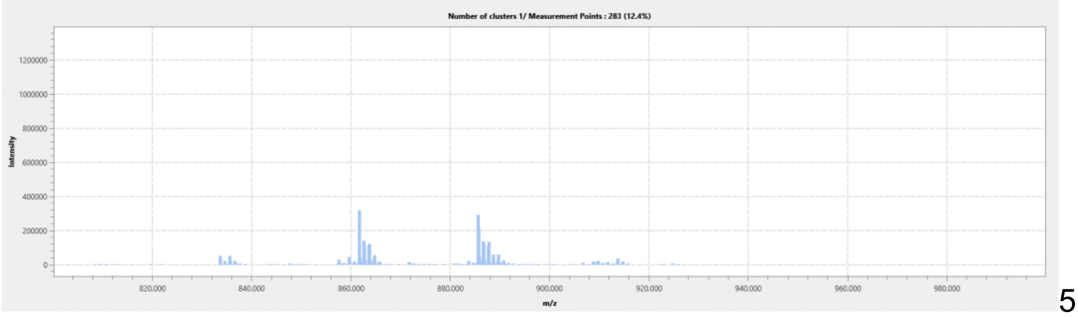
Number of clusters 1/ Measurement Points : 283 (12.4%)
Intensity
m/z
5

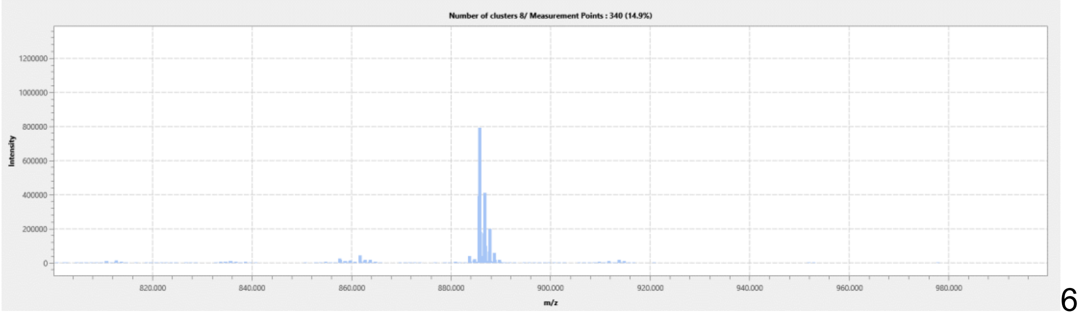
Number of clusters 8/ Measurement Points : 340 (14.9%)
Intensity
m/z
6

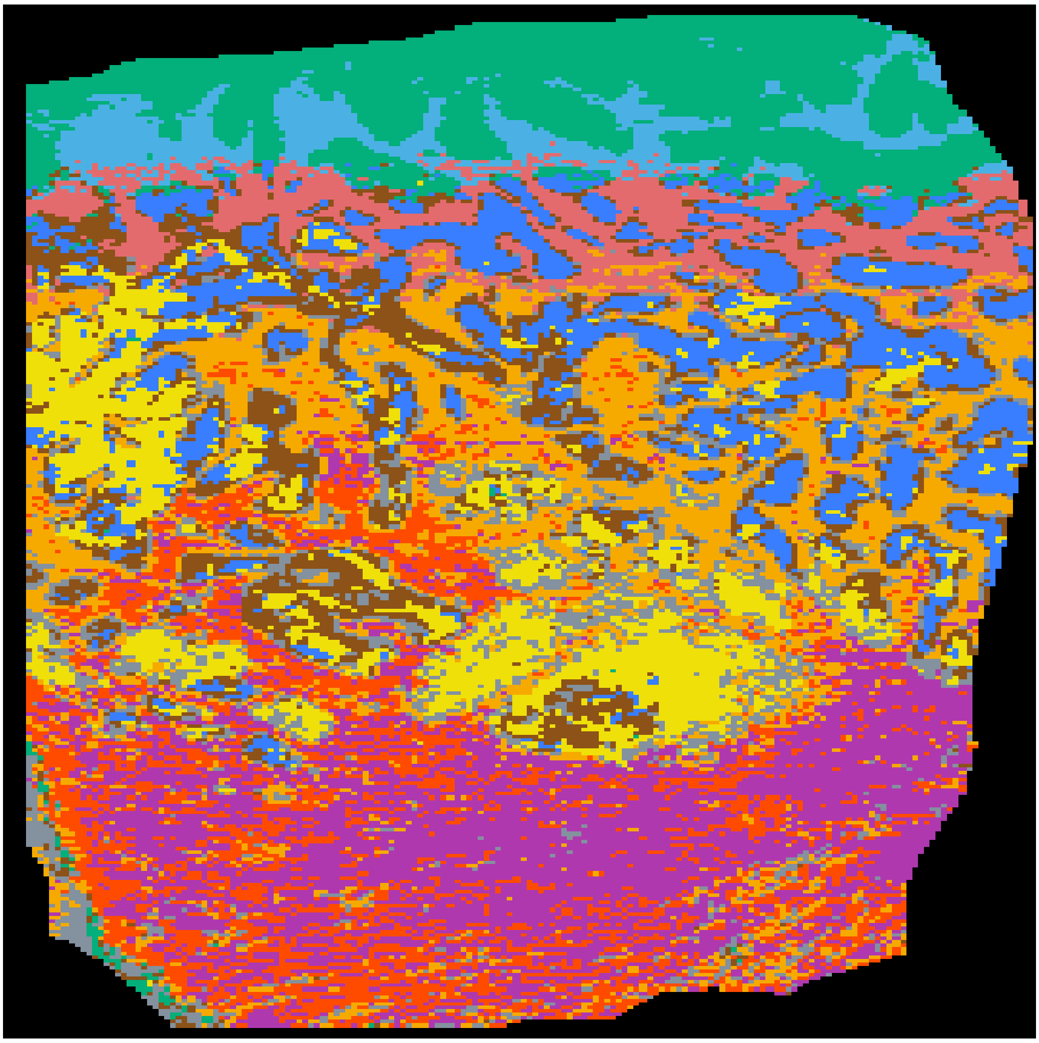

Figure S3. Sample spectra (Patient 7) and denary segmentation picture (800-1000 Da; negative mode)

Tumor pixel coloring (1-3): Green, Blue, Brick

Peritumor pixel coloring (4-6): Purple, Red, Yellow

Figures S1-S3 list the most common spectra of tumor and peritumor tissue and the colors assigned to them in Figures 3-5. Comparing the spectra of tumor and peritumor areas, it is evident that the intensities differ, indicating biochemical differences between tumors and the peritumor regions and suggesting that the IMS method could be used for this purpose.
